# The time is ripe: Natural variability of *MdNAC18.1* promoter plays a major role in fruit ripening

**DOI:** 10.1093/plcell/koaf004

**Published:** 2025-01-23

**Authors:** Christian Damian Lorenzo

**Affiliations:** Assistant Features Editor, The Plant Cell, American Society of Plant Biologists; Center for Plant Systems Biology, VIB, B-9052 Gent, Belgium; Department of Plant Biotechnology and Bioinformatics, Ghent University, B-9052 Gent, Belgium

Fruit ripening is a complex process that involves several biochemical and physiological changes, such as pigment increase, augmented softening, and flavor formation (Forlani et al. 2019). Several interconnected players regulate ripening. The phytohormone ethylene plays a pivotal role in initiating and determining ripening particularly in climacteric fruits like tomatoes and apples (Li et al. 2019). Members of the NAC family of plant-specific transcription factors have been shown to regulate ethylene production and affect fruit expansion and maturity (Zhang et al. 2024). Furthermore, studies in apple have reported associations between allelic variations in the coding sequence of a specific NAC, *MdNAC18.1*, and both harvest date and fruit firmness (Watts et al. 2023). This hints at an important role for this transcription factor in fruit maturity determination. However, the mechanism behind *MdNAC18.1* regulation of the ripening process and its further conservation in other climacteric fruits has not been further studied.

In new work, **Qianyu Yue, Yinpeng Xie, and collaborators (Yue et al. 2025)** found that the natural variability of *MdNAC18.1* plays an important role in defining ripening. Through a genome-wide association study of 204 late- and early-ripening apple cultivars, the researchers linked SNPs associated with ripening to *MdNAC18.1*. *MdNAC18.1* expression in early cultivars increased earlier after pollination compared with late ones, prompting further study of *MdNAC18.1* functionality. Due to the complexity of stable transformation in apple, the group resorted to tomato (another climacteric species) as a heterologous system to evaluate the role of *MdNAC18.1*. Tomato lines ectopically expressing *MdNAC18.1* had accelerated fruit ripening, evidenced by an earlier increase in ethylene production and faster rates of pigment accumulation relative to control plants. Similar results were obtained by transiently expressing *MdNAC18.1* in apple fruits, confirming that *MdNAC18.1* promotes ripening.

To investigate the molecular network associated with *MdNAC18.1*, a bulk RNA seq analysis was conducted comparing *MdNAC18.1OXs* with wild-type tomatoes. OX plants presented a marked upregulation of more than 70% of ripening-related markers, including genes involved in ethylene biosynthesis and signaling and pigment biosynthesis. Considering that NAC transcription factors can directly regulate gene expression, the authors performed chromatin immunoprecipitation qPCR assays as well as dual RENILLA / LUCIFERASE (REN/LUC) assays using the promoter regions of differentially expressed genes (DEGs) involved fruit ripening and ethylene synthesis. MdNAC18.1 was shown to directly bind to tomato DEG promoters. It also increased LUC levels when coexpressed with reporter constructs bearing the apple ortholog promoters of DEGs found in tomato, hinting at a conserved regulatory function.

Having strong evidence of a role for *MdNAC18.1* in fruit ripening, the authors wanted to determine the reason behind its divergent expression pattern in the different cultivars. Association studies of the early- and late-type promoters of *MdNAC18.1* revealed 3 variations strongly linked to ripening: 2 SNPs and a 58-bp insertion-deletion (InDel-58). Employing the same molecular toolbox, the group evaluated different artificial promoter layouts mimicking the early- or late-type promoters, as well as chimeric versions, swapping the variations among types to see how each could affect transcription. Although individual SNP changes contributed significantly to differences, the presence of InDel-58 was found to play a major role in regulating *MdNAC18.1* expression. Promoter variants with InDel-58 showed reduced LUC signal in reporter assays and delayed ripening when driving *MdNAC18.1* in both stable tomato lines and transient apple assays. Searching further in the flanking sequences around InDel-58, duplicated regulatory motifs could be found around the +58 variant. Using this region as bait in yeast 1-hybrid screenings yielded the detection of *MdAGL11* as a possible interactor. Reporter assays using the late and early promoter variants showed that upon coexpression with *MdAGL11*, LUC values were reduced for both type promoters but to a larger degree for the +58-bp variant. The results suggest that the duplicated element in late-ripening cultivars allows more binding *MdAGL11*, repressing *MdNAC18.1* expression. On the contrary, the deletion of the regulatory motif in early ripening cultivars leads to increased expression of *MdNAC18.1* and the upregulation of ripening-related genes (see Figure).

**Figure.**
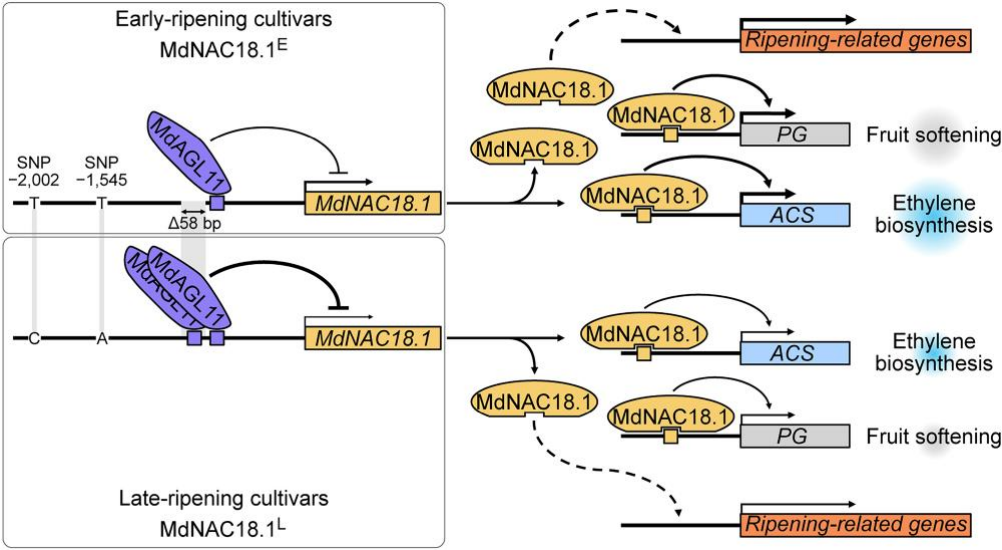
Proposed model of *MdNAC18.1* in regulating apple fruit ripening. MdNAC18.1 promotes apple fruit ripening by activating the ripening-related gene expression, including directly accelerating the expression of the fruit softening gene *PG* and ethylene biosynthesis gene *ACS*. Three variations within the promoter of *MdNAC18.1* are associated with fruit ripening, with InDel-58 playing a major role. In the early-ripening cultivars, InDel-58 is a deletion in *MdNAC18.1* promoter, which leads to attenuated repression of *MdNAC18.1* by *MdAGL11*, whose homolog is a negative regulator for fruit ripening. In the late-ripening cultivars, the InDel-58 is an insertion that results in 2 binding motifs of *MdAGL11*, thereby increasing the repression of *MdNAC18.1*. Reprinted from Yue et al. (2025), Figure 9.

The work of Yue et al. (2024) thus highlights how NAC-type transcription factors play an important role in the regulation of fruit ripening in climacteric species.

## Data Availability

No new data were generated or analysed in support of this research.

## Dive Curated Terms

The following phenotypic, genotypic, and functional terms are of significance to the work described in this paper:

NAC CHEBI: CHEBI:7421

fruit AmiGo: PO:0009001

## References

[koaf004-B1] Forlani S , Masiero S, Mizzotti C. Fruit ripening: the role of hormones, cell wall modifications, and their relationship with pathogens. J Exp Bot. 2019:70(11):2993–3006. 10.1093/jxb/erz112 30854549

[koaf004-B2] Li S , Chen K, Grierson D. A critical evaluation of the role of ethylene and MADS transcription factors in the network controlling fleshy fruit ripening. New Phytol. 2019:221(4):1724–1741. 10.1111/nph.15545 30328615

[koaf004-B3] Watts S , Migicovsky Z, Myles S. Large-scale apple GWAS reveals NAC18. 1 as a master regulator of ripening traits. Fruit Res. 2023:3(32). 10.48130/FruRes-2023-0032

[koaf004-B4] Yue et al A 58-1 bp InDel variant within the promoter region of MdNAC18.1 plays a Major role in fruit ripening. Plant Cell.2025.10.1093/plcell/koaf00739873675

[koaf004-B5] Zhang RX , Liu Y, Zhang X, Chen X, Sun J, Zhao Y, Zhang J, Yao JL, Liao L, Zhou H. Two adjacent NAC transcription factors regulate fruit maturity date and flavor in peach. New Phytol. 2024:241(2):632–649. 10.1111/nph.19372 37933224

